# Laparoscopic Repair for Recurrent Bilateral Inguinal Bladder Hernia following Bilateral Transabdominal Preperitoneal Repair

**DOI:** 10.1155/2018/4904093

**Published:** 2018-12-06

**Authors:** Akira Umemura, Takayuki Suto, Hisataka Fujuwara, Seika Nakamura, Hiroyuki Nitta, Takeshi Takahara, Yasushi Hasegawa, Akira Sasaki

**Affiliations:** ^1^Department of Surgery, Morioka Municipal Hospital, Japan; ^2^Department of Surgery, Iwate Medical University, Japan

## Abstract

**Introduction:**

Although a recurrent inguinal hernia is sometimes observed as a supravesical hernia, it is extremely rare to encounter a bilateral bladder sliding hernia recurrence. In this report, we describe an extremely rare case of a recurrent bilateral supravesical bladder hernia after bilateral transabdominal preperitoneal repair (B-TAPP).

**Case Presentation:**

A 69-year-old man visited our hospital with complaints of bilateral groin swelling and frequent voiding after B-TAPP. A plain CT revealed that the urinary bladder was herniating into the bilateral supravesical hernias. He underwent laparoscopic bilateral supravesical bladder hernia repair using a bladder takedown approach and median TAPP.

**Discussion:**

In Japan, the current mainstream method of hernioplasty is TAPP. However, an immature surgical technique and inadequate mesh placement may increase the risk of recurrent hernias. We successfully repaired this patient's recurrent bilateral supravesical bladder hernias laparoscopically.

**Conclusion:**

This rare condition (recurrent bilateral supravesical bladder hernias after B-TAPP) was successfully treated by using the bladder takedown approach and median TAPP. During surgical training and later in clinical practice, surgeons should master a surgical technique for this procedure in order to reduce recurrent hernias.

## 1. Introduction

It is well known that a recurrent inguinal hernia is sometimes observed as a supravesical hernia due to defective fixing of the mesh. However, it is extremely rare that a bladder sliding hernia occurs as a recurrent inguinal hernia [1] or bilateral recurrence. This paper describes our extremely rare experience using laparoscopic repair for the recurrence of a bilateral inguinal bladder hernia following bilateral transabdominal preperitoneal repair.

## 2. Case Presentation

**Figure 1:**
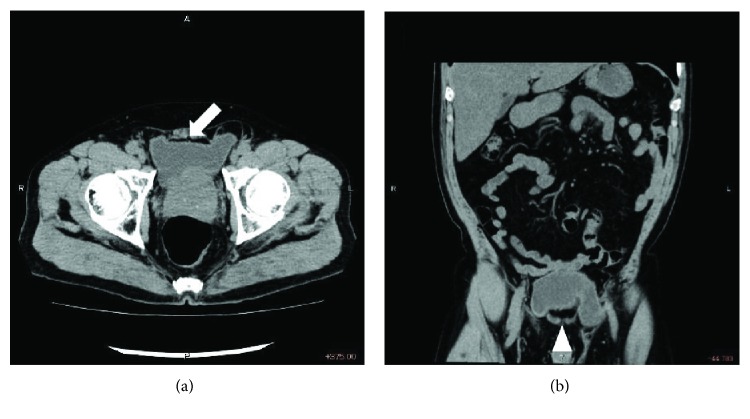
Preoperative CT findings. (a) An axial slice showed a typical “Micky Mouse” shape (white arrow). (b) A coronal slice also revealed bilateral urinary bladder sliding into hernia orifices (white triangle).

A 69-year-old man who suffered from pain and swelling in the bilateral groin and from frequent voiding visited our clinic. He had undergone bilateral transabdominal preperitoneal repair (B-TAPP) three years earlier at another hospital. During his physical examination, bilateral inguinal swelling was observed before urination, and he complained about micturition when we tried to reintroduce the hernia contents. A CT scan revealed that his urinary bladder was herniating into the bilateral inguinal hernias with a typical “Mickey Mouse” shape (Figures 1(a) and 1(b)). From these findings, we diagnosed a recurrent bilateral bladder hernia after B-TAPP. Then, we preoperatively planned to employ the bladder takedown approach to pull the urinary bladder from the orifice and to perform median TAPP.

**Figure 2:**
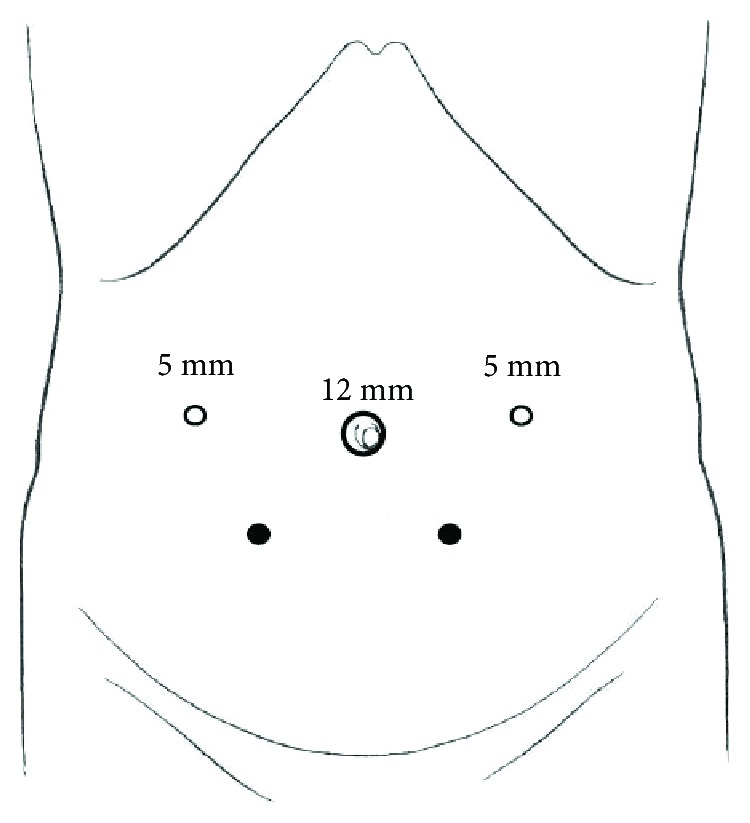
Trocar placements. Two 5 mm trocars were placed on the cranial side of the umbilicus (white rounds) compared to usual TAPP (black rounds).

**Figure 3:**
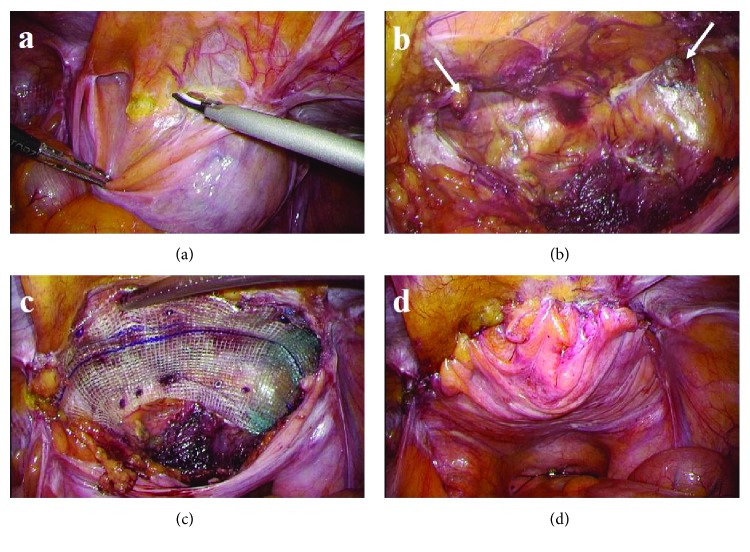
Surgical procedures. (a) The peritoneum was incised at the ventral side of the urinary bladder. (b) Hernia orifices were exposed by a bladder takedown approach (arrows). (c) A self-fixating mesh was also fixed with Cooper's ligament and rectus sheath. (d) The preperitoneal space was sutured and closed to screen the mesh.

With the patient in the supine position under general anesthesia, we inserted three trocars as shown in Figure 2. Trocar placements were on the cranial side of the umbilicus compared to usual TAPP. At first, we confirmed that the lateral side of the mesh was appropriately covered by the prior mesh and that there were no recurrent indirect hernias. Then, we incised the peritoneum on the ventral side of the urinary bladder and took down the bladder to expose the hernia orifices (Figure 3(a)). Both hernia orifices were located inside of both medial umbilical folds; therefore, we intraoperatively diagnosed a recurrent bilateral supravesical bladder hernia with no lateral recurrences (Figure 3(b)). We introduced a laparoscopic self-fixating mesh (ProGrip™, Medtronic Inc., Minneapolis, MN, USA) into the peritoneal cavity and unrolled the mesh as both hernia orifices were covered. The mesh was fixed with Cooper's ligament and the rectus sheath by a mesh fixation device (AbsorbaTack™, Medtronic Inc., Minneapolis, MN, USA) to keep it from slipping (Figure 3(c)). Finally, we sutured the urinary bladder with the ventral peritoneum to close the preperitoneal space and to screen the mesh using a barbed suture device (V-Loc™ 180, Medtronic Inc., Minneapolis, MN, USA) (Figure 3(d)). The operative time was 132 minutes, and the total blood loss was 2 mL. We included only median mesh repair for bilateral recurrent inguinal hernias as the name of the median TAPP procedure.

**Figure 4:**
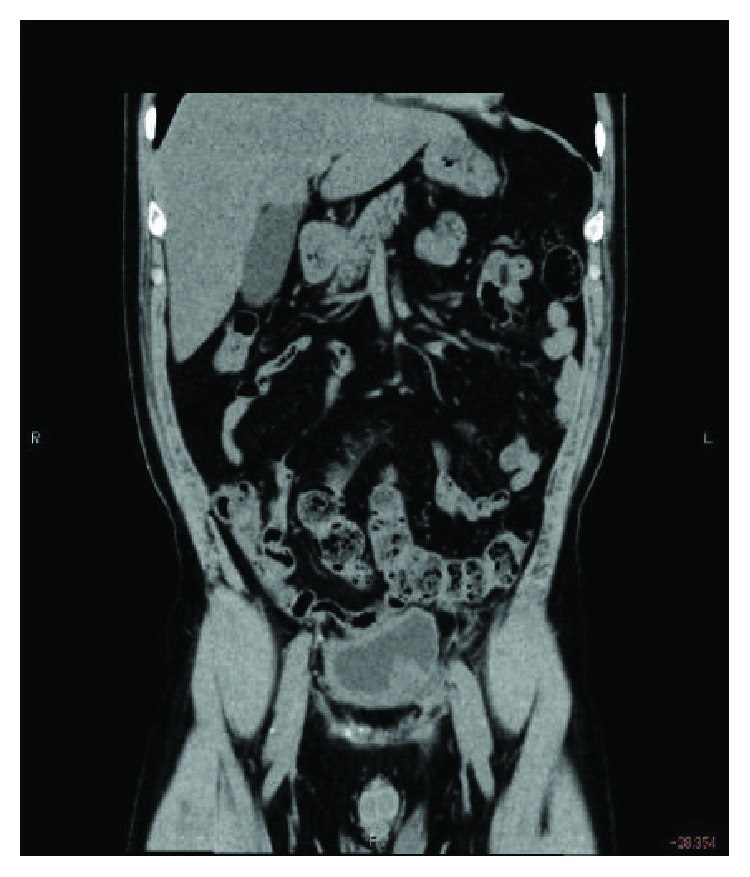
Postoperative CT findings. A coronal slice revealed that appropriate repair for bilateral bladder sliding hernia was performed.

The patient was discharged on postoperative day 3 without any complications, and his preoperative symptoms disappeared. A postoperative CT showed no recurrence of the bilateral supravesical bladder hernias (Figure 4).

## 3. Discussion

The incidence of the urinary bladder within sliding hernias is 5.6–12.5% and less than 1% among all hernias [1, 2]. Patients with a bladder sliding inguinal hernia often present with obstructive urinary symptoms such as scrotal swelling, urination, incomplete voiding, hydronephrosis, and acute renal failure [3]. In our case, frequent voiding was the main symptom due to bladder herniation. To the best of our knowledge, some case reports about unilateral and bilateral bladder hernias were found [4, 5]; however, this is the first case report concerning a recurrent bilateral inguinal bladder hernia following B-TAPP and repaired by median TAPP.

Laparoscopic repairs for inguinal hernias, such as TAPP and totally extraperitoneal repair (TEP), have become mainstream in the surgical profession. However, unskilled TAPP and TEP may increase the recurrence rate compared to an open approach [6]. In addition, recurrent inguinal hernias sometimes present complicated patterns, such as what we experienced. In principle, the anterior approach is recommended for recurrent inguinal hernias owing to the difficulty of laparoscopic procedures and high recurrence rates [7]. At first, although we planned to perform mesh plug repair via the anterior approach, we changed to laparoscopic repair using a bladder takedown approach and median TAPP because we were worried that bilateral mesh plugs might penetrate the urinary bladder owing to the compartmental formation of the preperitoneal space after B-TAPP [8]. For these reasons, we chose the challenging median TAPP procedure.

A bladder takedown approach is usually used in laparoscopic suprapubic incisional hernia repair and is called the transabdominal partial extraperitoneal technique [9]. Since no lateral recurrences were observed in this case, both supravesical hernia orifices were essentially equivalent to suprapubic incisional hernias; therefore, we employed this technique. However, laparoscopic self-fixating mesh differs from meshes used in ventral hernia repair in that antiadhesive material is not coated on the side of the abdominal cavity because a mesh has to be screened by peritoneal closure so as not to have contact with abdominal organs.

## 4. Conclusion

A sliding hernia that includes the urinary bladder is a rare occurrence, comprising less than 1% of all hernia cases [1, 2]; moreover, a recurrent, bilateral, and supravesical bladder hernia is an extremely rare case. The bladder takedown approach was appropriate for exposing the hernia orifices, and median TAPP was a reasonable and effective procedure for this patient. During surgical training and later in clinical practice, surgeons should master a surgical technique for this procedure in order to reduce recurrent hernias [6].
